# Establishment and Maintenance of a Digital Therapeutic Alliance in People Living With Negative Symptoms of Schizophrenia: Two Exploratory Single-Arm Studies

**DOI:** 10.2196/64959

**Published:** 2025-01-27

**Authors:** Cassandra Snipes, Cornelia Dorner‑Ciossek, Brendan D Hare, Olya Besedina, Tim Campellone, Mariya Petrova, Shaheen E Lakhan, Abhishek Pratap

**Affiliations:** 1 Click Therapeutics Inc. New York, NY United States; 2 Boehringer Ingelheim International GmbH Ingelheim am Rhein Germany; 3 Boehringer Ingelheim Pharmaceuticals, Inc. Ridgefield, CT United States

**Keywords:** therapeutic alliance, digital working alliance, experiential negative symptoms, schizophrenia, digital therapeutics, digital literacy

## Abstract

**Background:**

Evidence-based digital therapeutics represent a new treatment modality in mental health, potentially providing cost-efficient, accessible means of augmenting existing treatments for chronic mental illnesses. CT-155/BI 3972080 is a prescription digital therapeutic under development as an adjunct to standard of care treatments for patients 18 years of age and older with experiential negative symptoms (ENS) of schizophrenia. Individual components of CT-155/BI 3972080 are designed based on the underlying principles of face-to-face treatment. A positive therapeutic alliance between patients and health care providers is linked with improved clinical outcomes in mental health. Likewise, establishing a similar therapeutic alliance with a digital therapeutic (ie, digital working alliance [DWA]) may be important for engagement and treatment effectiveness of this modality.

**Objective:**

This study aimed to investigate the establishment and maintenance of a DWA between a beta version of CT-155/BI 3972080 (CT-155 beta) and adults with ENS of schizophrenia.

**Methods:**

Two multicenter, exploratory, single-arm studies (study 1: CT-155-C-001 and study 2: CT-155-C-002) enrolled adults with schizophrenia and ENS receiving stable antipsychotic medication (≥12 weeks). Participants had access to CT-155 beta and were presented with daily in-app activities during a 3-week orientation phase that included lessons designed to facilitate building of a DWA. In study 2, the 3-week orientation phase was followed by an abbreviated active 4-week phase. Digital literacy at baseline was evaluated using the Mobile Device Proficiency Questionnaire (MDPQ). The mobile Agnew Relationship Measure (mARM) was used to assess DWA establishment after 3 weeks in both studies, and after 7 weeks in study 2 to assess DWA maintenance. Participant safety, digital literacy, and correlations between negative symptom severity and DWA were assessed in both studies.

**Results:**

Of the enrolled participants, 94% (46/49) and 86% (43/50) completed studies 1 and 2, respectively. Most were male (study 1: 71%, 35/49; study 2: 80%, 40/50). The baseline digital literacy assessed through MDPQ score was comparable in both studies (study 1: mean 30.56, SD 8.06; study 2: mean 28.69, SD 8.31) indicating proficiency in mobile device use. After 3 weeks, mARM scores (study 1: mean 5.16, SD 0.8; study 2: mean 5.36, SD 1.06) indicated that a positive DWA was established in both studies. In study 2, the positive DWA established at week 3 was maintained at week 7 (mARM: mean 5.48, SD 0.97). There were no adverse events (AEs) in study 1, and 3 nonserious and nontreatment-related AEs in study 2.

**Conclusions:**

A positive DWA was established between participants and CT-155 beta within 3 weeks. The second 7-week study showed maintenance of the DWA to the end of the study. Results support the establishment and maintenance of a DWA between adults with ENS of schizophrenia and a beta version of CT-155/BI 3972080, a prescription digital therapeutic under development to target these symptoms.

**Trial Registration:**

Clinicaltrials.gov NCT05486312; https://clinicaltrials.gov/study/NCT05486312

## Introduction

The therapeutic alliance was defined by Bordin and colleagues in 1979 as a measure of the quality of the relationship between a patient and health care provider, and its pivotal influence on clinical outcomes is well documented and supported [1-3]. The following three main components constitute the therapeutic alliance: (1) the patient-provider bond, (2) a collaborative approach to assignment of therapeutic tasks, and (3) agreement on therapeutic goals [1]. In face-to-face therapy, the quality of this working alliance predicts clinical outcomes independently of the type of implemented psychotherapy or assessed outcome measures [2-6]. Several studies suggest that there is an association between a positive therapeutic alliance and improved outcomes in people with mental illness, including schizophrenia. For example, there is evidence that maintaining a positive alliance is associated with adherence to schizophrenia medication [7,8], that a positive alliance predicts improved overall psychotic symptomatic outcomes [9,10], and that strong alliances are associated with better treatment engagement and improvements in schizophrenia symptoms [11].

Schizophrenia is a chronic debilitating psychiatric disorder that occurs in approximately 1 in every 300 people worldwide [12]. Symptoms generally emerge in late adolescence or early adulthood and are categorized as positive, negative, or cognitive [13,14]. Positive symptoms, such as delusions, hallucinations and disorganized thinking, represent an excess or distortion of normal functions, and tend to relapse and remit. Negative symptoms involve reductions or absences in normal emotional and social functioning. Cognitive symptoms affect memory, attention and executive function. Both negative and cognitive symptoms being chronic in nature contribute substantially to the significant emotional and socioeconomic burden associated with schizophrenia [15-18]. Experiential negative symptoms (ENS) are a key subset of negative symptoms characterized by deficits in pleasure (anhedonia), motivation (avolition), and social interest (asociality), and have a profound impact on functional outcomes [19,20].

The American Psychiatric Association practice guidelines for the treatment of people with schizophrenia highlight the benefits of evidence-based psychosocial treatments, social skills training and psychoeducation as adjunct therapies to medication [21]. To date there is no treatment approved by the US Food and Drug Administration (FDA) for negative symptoms. Despite promising evidence for psychological approaches including cognitive behavioral therapy and cognitive remediation in treating negative symptoms [22-24], people with schizophrenia face significant barriers to engaging in psychosocial therapy. These include symptom-related barriers, stigma and social isolation, and insufficient health care resources [25-27], all of which may contribute to a low uptake of psychosocial therapy in people with schizophrenia [27-30].

Digital therapeutics (DTx) have the potential to bridge this gap by broadening access to adjunctive psychosocial therapies and facilitating greater frequency of exposure in a way that is flexible and convenient for those receiving treatment [31,32]. DTx are defined by the International Organization for Standardization as any evidence-based “health software intended to treat or alleviate a disease, disorder, condition, or that has a demonstrable positive therapeutic impact on a patient’s health” [33]. For DTx intended as treatments for vulnerable populations such as those with serious mental illness, it is particularly important that they are regulated, rigorously tested, and found to have proven effectiveness and strong safety profiles. Recent studies evaluating novel DTx in the form of smartphone apps have shown this modality to be acceptable and feasible to implement in individuals with chronic psychiatric disorders such as schizophrenia [34-37]. While there is evidence to suggest that the therapeutic alliance can be established and maintained in remote online settings of psychotherapy [4,38,39] it is not fully understood if individuals with severe mental illness, such as schizophrenia, can establish and maintain a therapeutic alliance with a DTx [40-43]. Importantly, while recent studies in people with schizophrenia evaluated the feasibility of DTx they did not assess the establishment of a therapeutic alliance [34,35,44].

We conducted 2 independent studies to assess the establishment and maintenance of a digital working alliance (DWA) between adults with ENS of schizophrenia and a beta version of CT-155/BI 3972080 (hereafter referred to as CT-155 beta), a novel software accessible on mobile phones under development to treat ENS of schizophrenia adjunctive to standard of care. In December 2023, CT-155/BI 3972080 was granted breakthrough device designation by the FDA [45]. In the studies reported here, we assessed whether it is feasible to establish and maintain a positive DWA between people living with ENS of schizophrenia and CT-155 beta, and if the strength of the DWA correlates or differs according to severity of negative symptoms, age, or race.

## Methods

### Study Design

**Figure 1:**
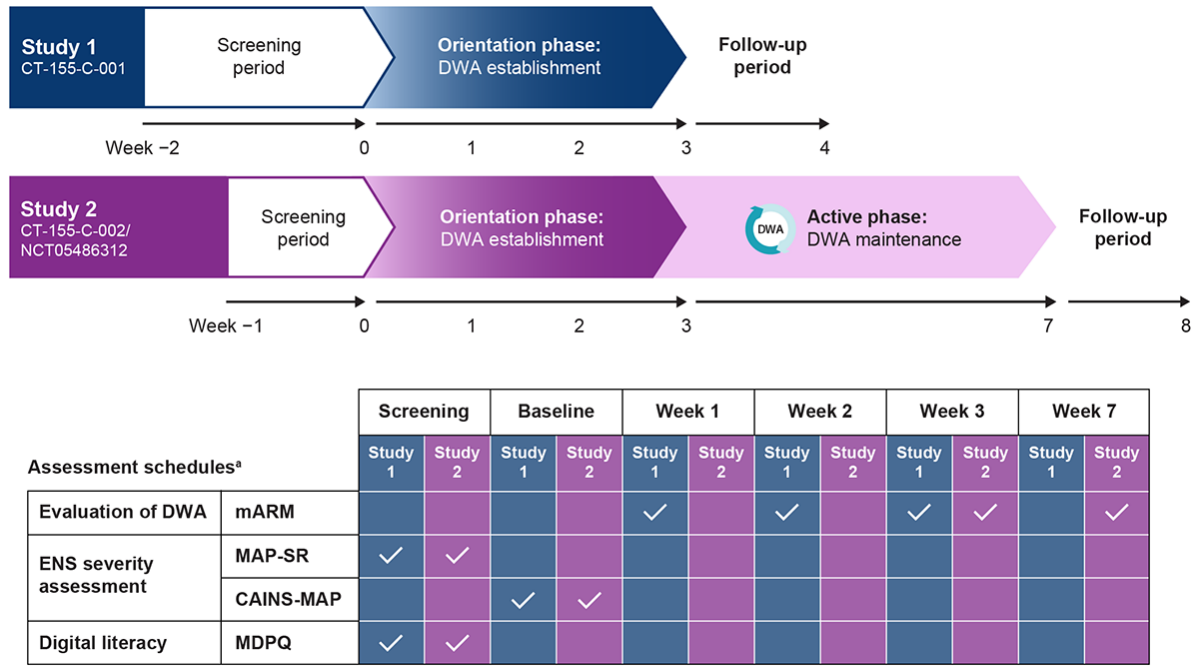
Study design. Two independent single-arm, multicenter, exploratory studies (study 1: CT-155-C-001, study 2: CT-155-C-002/NCT05486312) were conducted across 10 sites in the United States. ^a^Schedules for the key assessments are presented. CAINS-MAP: Clinical Assessment Interview for Negative Symptoms–Motivation and Pleasure; DWA: digital working alliance; ENS: experiential negative symptoms; MAP-SR: Motivation and Pleasure–Self Report; mARM: mobile Agnew Relationship Measure; MDPQ: Mobile Device Proficiency Questionnaire.

Two independent single-arm, multicenter, exploratory studies (study 1: CT-155-C-001 and study 2: CT-155-C-002/NCT05486312) were conducted across 10 sites in the United States. Patient informed consent was obtained at an initial in-person screening visit, during which site personnel assisted with the download and setup of CT-155 beta onto participants’ personal smartphone devices. Eligibility was confirmed and CT-155 beta was activated at an in-person baseline visit on Day 1. Both studies comprised a screening period, a treatment period, and a follow-up period (Figure 1). The treatment period comprised a 3-week orientation phase in study 1 and study 2, with an additional 4-week active phase in study 2 (further details in Intervention section). Both studies concluded with a follow-up period during which a remote teleconference follow-up visit occurred (Figure 1).

### Participants

Eligible participants were 18-64 years old (study 1) and ≥18 years old (study 2) with a primary diagnosis of schizophrenia for at least 1 year as per the *ICD-10* (*International Classification of Diseases, Tenth Revision*; study 1) [46], and the *DSM-5* (*Diagnostic and Statistical Manual of Mental Disorders* [Fifth Edition]; studies 1 and 2) [13], with moderate-to-severe ENS (defined as a score of ≤30 on the Motivation and Pleasure–Self Report [MAP-SR]) [47], and receiving a stable dose of a maximum of 2 different antipsychotic medications for at least 12 weeks before screening. Exclusion criteria included a *DSM-5* or *ICD-10* diagnosis other than schizophrenia, or a substance or alcohol use disorder, excluding caffeine and nicotine. People receiving clozapine, haloperidol, or who had received psychotherapy within 12 weeks of screening were also excluded. People with active prominent positive symptoms that, in the opinion of the investigator, would preclude effective engagement in treatment for negative symptoms were also excluded. The complete list of eligibility criteria for both studies is included in Table S1 in Multimedia Appendix 1.

### Intervention

CT-155/BI 3972080 is a novel software accessible on mobile devices, designed to treat the ENS of schizophrenia and under development for use adjunctive to standard of care treatments for schizophrenia. As such CT-155/BI 3972080 is currently FDA regulated as an investigational device, more specifically, a software as a medical device [48]. The development of the software was informed by an iterative patient-centered design process, which consisted of 2 phases during which feedback was captured from 4 peer support specialists with lived experiences of schizophrenia, and 15 patient panel participants with a diagnosis of schizophrenia (refer to Supplementary Methods in Multimedia Appendix 1 for further details).

**Figure 2:**
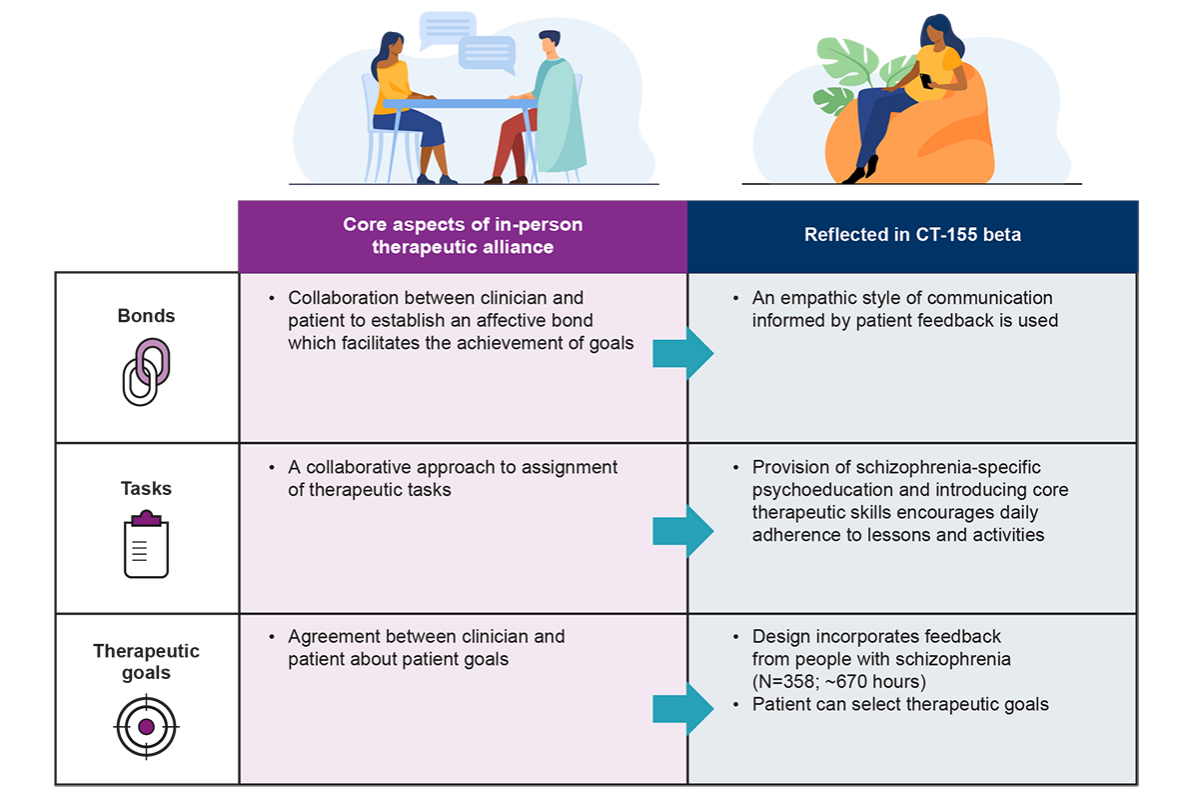
CT-155 beta captures aspects of in-person therapeutic alliance. Individual components of CT‑155 beta are designed based on the underlying principles of face-to-face treatment. These therapeutic techniques are intended to work synergistically to treat ENS and are supported by personalized engagement functionality that works in concert with the core therapeutic mechanisms. DWA: digital working alliance; ENS: experiential negative symptoms.

CT-155 beta has 3 core phases. First, the 3-week orientation phase in each study was designed based on the core features of in-person therapeutic alliance to encourage daily adherence to lessons and activities by providing schizophrenia-specific psychoeducation and introducing core therapeutic skills (Figure 2) [41]. An empathic style of communication informed by patient feedback, provision of schizophrenia-specific psychoeducation, and introducing core therapeutic skills are patient-centric features incorporated into CT-155. Like therapists getting to know their patient, this phase focuses on DWA establishment through asking questions and rapport building, followed by collaborative treatment approach and goal definition. These patient-centric features that support DWA are subsequently integrated into all therapeutic components to optimize the experience by infusing an interactive, empathic, knowledgeable, and personally meaningful nature to the in-app experience that builds confidence for achieving treatment goals and is critical to treatment outcome. Second, study 2 included an active phase, during which participants receive access to CT-155 beta for an additional 4 weeks after the 3-week orientation phase (Figure 1), designed to reinforce orientation phase components and introduce adaptive goal setting [49,50]. The active phase comprises evidence-based digitized therapeutic techniques leveraging the underlying principles of face-to-face treatment [51-54]. Examples of the therapeutic techniques used include adaptive goal setting [49,50] and social skills training [21]. Adaptive goal setting is focused on delivering interventions known to target negative symptoms in important functional areas (ie, social items, work and school items, and recreation items). Finally, during a brief follow-up phase, participants from both studies complete qualitative exit interviews to help understand the user experience and inform further CT-155/BI 3972080 development.

### Protocol Deviations

In study 1, 12 major protocol deviations occurred with 10 participants (Table S2 in Multimedia Appendix 1). Deviations included assessment performed outside of protocol window (n=6), CT-155 beta was uninstalled (n=2), failed inclusion criteria (n=2), and visit occurred outside of protocol window (n=1). One participant with failed inclusion deviation was deemed ineligible and terminated the study early. One participant had 3 major deviations (uninstalled CT-155 beta before week 4, schizophrenia diagnosis confirmed <1 year before enrollment, and antipsychotic added <12 weeks before screening) was included in the intent-to-treat-population but excluded from the per protocol population. In study 2, 1 major protocol deviation occurred with 1 participant (visit outside protocol window; Table S2 in Multimedia Appendix 1).

### Assessments

#### Patient Digital Literacy

The digital literacy of participants at baseline was assessed using the self-administered Mobile Device Proficiency Questionnaire (MDPQ). While developed for assessing mobile proficiency in older adults, the MDPQ has also been used in health studies to assess proficiency in individuals with subjective cognitive complaints and those with cirrhosis [55-57]. The MDPQ consists of 46 items rated on a 5-point Likert scale and assesses seven aspects of digital literacy: (1) mobile device basics, (2) communication, (3) data and file storage, (4) internet, (5) entertainment, (6) privacy, and (7) troubleshooting and software management [55]. Participants were asked to rate their ability to perform specific digital tasks on a scale of 1 (never tried it) to 5 (very easily performed). Subscale scores were obtained by averaging responses within each subscale and overall scores were calculated as the sum of mean subscale scores. The overall scores fall in a range of 8 (low) to 40 (great ability to perform a number of tasks with a mobile device). Internal reliability of the MDPQ was demonstrated with a Cronbach α score of 0.975.

#### Experiential Negative Symptom Severity Assessment

Participants completed the MAP-SR at screening. The MAP-SR is a 15-item validated self-administered tool that assesses the motivation and pleasure domain of negative symptoms in people with psychotic disorders and scores are rated on a 5-point Likert scale, where low scores reflect greater severity [47]. In addition, the Clinical Assessment Interview for Negative Symptoms–Motivation and Pleasure (CAINS-MAP) assessment was conducted at baseline in both studies, and at week 7 in study 2, to evaluate ENS symptom severity. The CAINS-MAP is a validated 13-item clinician-administered interview-based assessment with items scored on a 4-point scale, with lower scores indicating lower negative symptom severity [58].

#### Digital Working Alliance Assessment

The DWA was assessed using the self-administered 25-item mobile Agnew Relationship Measure (mARM) questionnaire. The mARM is a measure used to evaluate the DWA of mobile health interventions across 5 therapeutic alliance domains: bonding, partnership, confidence, openness, and initiative [59]. It provides an alliance measure with good content and face validity suitable for assessing alliance with DTx [59]. The mARM includes five subscales to represent the dimensions of the DWA: (1) the bond subscale focuses on the perceived acceptance, support, friendliness, and understanding the patient develops with the app, (2) the partnership subscale measures the extent of the perceived collaboration of the patient with the therapeutic tasks, (3) the confidence subscale focuses on patient optimism and their belief in the competency of the app, (4) the openness subscale assesses the perceived freedom to disclose personal concerns without fear of judgment or embarrassment, and (5) the client initiative subscale focuses on the client willingness to take responsibility for their treatment progress.

The mARM was administered at week 1, week 2, and week 3 in study 1 to assess the establishment of the DWA during the orientation phase. In study 2, it was administered at week 3 to assess DWA establishment and again at week 7 to evaluate the maintenance of DWA during the abbreviated active phase. The overall mARM score is calculated at each time point as the mean of all subscale scores and is interpreted along a Likert scale ranging from 1 (strongly disagree) to 7 (strongly agree), with 4 equating to “neutral,” and scores higher than the neutral midpoint suggesting a positive DWA [59-61]. The potential association between various factors including age, race, and ENS severity, and the DWA was also assessed.

#### Safety

Safety was assessed by recording adverse events (AEs), serious AEs, adverse device effects (ADEs), serious ADEs, and discontinuations from the study due to AEs, from signing of informed consent through to the end of study period.

### Sample Size and Statistical Analyses

Both studies were exploratory in nature and sample size and analyses were selected for optimal assessment of DWA establishment and maintenance between schizophrenia patients and CT-155 beta. Data presented are from the intent-to-treat population, defined as all participants who were enrolled in each study to receive treatment with CT-155 beta. Descriptive statistics were evaluated for all study variables. To determine if DWA was related to ENS, age, or engagement, correlation analyses between mARM scores and baseline CAINS-MAP, age at screening, and session completion were performed using a Spearman rank-order correlation analysis and correlation coefficients were calculated together with the 95% 2-sided confidence intervals. To investigate effects of race, variance of the mean overall mARM score between Black or African American and participants of all other races were compared using an *F* test. To assess whether digital literacy was related to ENS and engagement, correlation analyses between baseline MDPQ scores and CAINS-MAP and session completion scores were also performed using a Spearman rank-order correlation analysis. Engagement was assessed using the number of sessions completed during the study whereby a session was defined as any interaction within CT-155 beta for >60 seconds.

### Ethical Considerations

The study protocols and study materials were reviewed by the Western Copernicus Group independent institutional review board (approval numbers: 20215315 [study 1] and 20220609 [study 2]). The studies were conducted in compliance with the respective clinical study protocols, in accordance with the principles of the Declaration of Helsinki [62], the International Council for Harmonisation of Good Clinical Practice [63], and applicable US regulatory requirements. Patient informed consent was obtained from all participants. Participants were deidentified and assigned a unique identifier to protect personal data and privacy throughout both studies. Throughout both studies, a study team maintained compliance with applicable laws and accepted security standards, including the Health Insurance Portability and Accountability Act of 1996, the U.S. National Institute of Standards and Technology Framework, and the Service Organization Control Type 2 controls. The compliance program was designed to (1) ensure the security, confidentiality, integrity, and availability of the assets and other sensitive information that was collected, used, and maintained; (2) protect against any anticipated threats or hazards to the security, integrity, or availability of such information; (3) maintain information security controls that were appropriate to Click Therapeutics size, scope, and business; (4) maintain safeguards and controls to protect information from loss, theft, destruction, unauthorized manipulation, disclosure, or unavailability. Participants were offered up to US $465 and US $735 for partaking in study 1 and study 2, respectively. Incentive values reflect fair market value; variance in incentive values is reflective of the differing observation periods between the 2 studies.

## Results

### Patient Demographics and Baseline Characteristics

**Figure 3:**
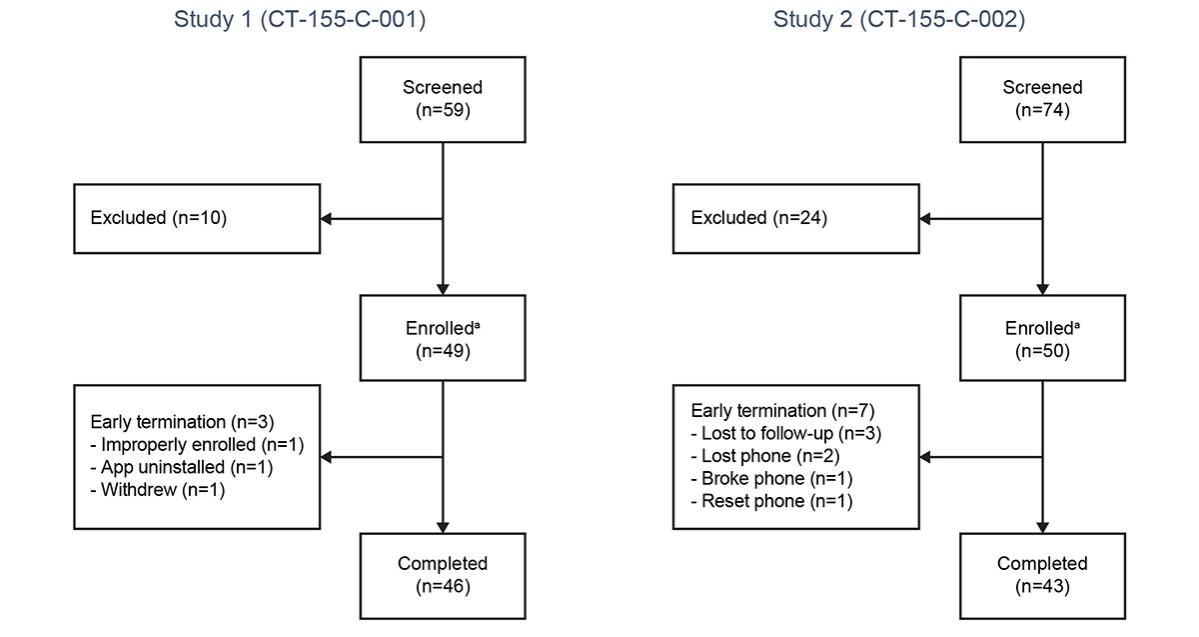
Participant disposition. ^a^Intent-to-treat population.

In study 1, a total of 49 participants were enrolled and 46 (94%) completed the study. In study 2, a total of 50 participants were enrolled and 43 (86%) completed the study. Participant disposition is presented in Figure 3. The median age of enrolled participants was 46 (range 18-64) years in study 1 and 53.5 (range 23-64) years in study 2. Of those enrolled, most were male (study 1: 35/49, 71%; study 2: 40/50, 80%), and more than half were Black or African American (study 1: 27/49, 55%; study 2: 29/50, 58%). The majority never attended college (study 1: 31/49, 63%; study 2: 32/50, 64%) and most had a self-reported annual income of <US $25,000 (study 1: 46/49, 94%; study 2: 47/50, 94%; Table 1). The median MAP-SR score at screening was 25.0 (IQR 17.0-28.0) in study 1 and 15.0 (IQR 7.0-23.0) in study 2. At baseline, the median overall CAINS-MAP score was 2.4 (IQR 2.0-2.9) in study 1 and 2.6 (IQR 1.6-2.9) in study 2. Baseline demographics and characteristics of study 2 participants are also being reported in another manuscript reporting separate outcomes.

**Table 1:** Baseline demographics and characteristics.

Baseline characteristics			Total enrolled in study 1 (n=49)		Total enrolled in study 2 (n=50)
**Sex, male, n (%)**			35 (71)		40 (80)
**Race, n (%)**					
	Black or African American	27 (55)		29 (58)	
	White	21 (43)		15 (30)	
	Asian	1 (2)		3 (6)	
	American Indian or Alaska Native	0 (0)		1 (2)	
	Other	0 (0)		2 (4)	
**Ethnicity, n (%)**					
	Hispanic or Latino	13 (27)		12 (24)	
	Not Hispanic or Latino	36 (74)		38 (76)	
**Education, n (%)**					
	Less than high school	10 (20)		7 (14)	
	High school	21 (43)		25 (50)	
	Some college	10 (20)		12 (24)	
	College degree	8 (16)		6 (12)	
**Annual income (US $), n (%)**					
	<25,000	46 (94)		47 (94)	
	25,000-49,999	3 (6)		3 (6)	
**Screening assessments**					
	MAP-SR^a^, median (IQR)	25.0 (17.0-28.0)		15.0 (7.0-23.0)	
**Baseline assessments**					
	MDPQ^b^, median (IQR)	32.0 (23.2-39.1)		30.1 (21.4-35.5)	
	CAINS-MAP^c^ overall score, median (IQR)	2.4 (2.0-2.9)		2.6 (1.6-2.9)	

^a^MAP-SR: Motivation and Pleasure scale–Self Report.

^b^MDPQ: Mobile Device Proficiency Questionnaire.

^c^CAINS-MAP: Clinical Assessment Interview for Negative Symptoms Motivation and Pleasure subscale.

The median overall MDPQ score was comparable in both studies at baseline (study 1: 32.01, IQR 23.20-39.07; study 2: 30.10, IQR 21.36-35.46), indicating proficiency in the use of mobile devices before accessing CT-155 beta. A summary of MDPQ overall and subscale scores is presented in Table 2. Overall, MDPQ subscales scores revealed proficiency across most operations from the basic task of “turning the device on and off” and communication tasks, such as “open emails” to the more advanced tasks such as those related to privacy “Setting up a password to lock/unlock the device,” and tasks related to the troubleshooting and software management, such as “Restarting the device when it is frozen, or not working right.” However, participants in both studies were slightly less proficient with data and file storage tasks, such as “Transfer information (files such as music, pictures, documents) from portable device to computer” (Table 2). There was no correlation between MDPQ and baseline CAINS-MAP in study 1 (ρ=0.01, 95% CI –0.31 to 0.34) and study 2 (ρ=–0.01, 95% CI –0.29 to 0.28). Furthermore, there was no correlation between MDPQ and degree of participant engagement with CT-155 beta (number of completed sessions) in study 1 (ρ=0.00, *P*=.99) and study 2 (ρ=–0.18, *P*=.22).

**Table 2:** Mobile Device Proficiency Questionnaire overall and subscale scores at baseline in study 1 and study 2.

MDPQ^a^ scores			Study 1, median (IQR) (n=42)		Study 2, median (IQR) (n=49)
Overall			32.01 (23.20-39.07)		30.10 (21.36-35.46)
**Subscale scores**					
	Mobile device basics	4.61 (4.00-5.00)		4.44 (3.67-4.89)	
	Communication	3.94 (2.33-5.00)		3.67 (2.56-4.56)	
	Data and file storage	3.00 (1.67-5.00)		3.00 (1.00-4.00)	
	Internet	4.25 (3.38-5.00)		3.88 (2.50-4.88)	
	Calendar	4.00 (2.67-5.00)		4.00 (2.33-4.67)	
	Entertainment	4.70 (3.40-5.00)		4.20 (3.40-5.00)	
	Privacy	4.00 (3.00-5.00)		4.00 (2.50-5.00)	
	Troubleshooting and software management	4.00 (2.75-5.00)		4.00 (2.60-4.80)	

^a^MDPQ: Mobile Device Proficiency Questionnaire.

### Digital Working Alliance Summary

The mean overall mARM score in study 1 was 5.15 (SD 0.74) indicating establishment of a positive DWA by week 1 that did not significantly change by week 3, 5.16 (SD 0.77). Similarly, in study 2, participants also formed a positive DWA by week 3, with a mean overall mARM score of 5.36 (SD 1.06; Table 3). Furthermore, the positive DWA in study 2 was maintained through the subsequent 4-week treatment period with mean overall mARM score of 5.48 (SD 0.97) at week 7 (Table 3). The individual mARM subscale scores (Bond, Partnership, Confidence, Openness, and Client initiative) remained consistent and above “neutral” (score of 4) at the end of the DWA and core-skills-building phase at week 3 in both studies demonstrating that participants successfully formed a positive DWA with CT-155 beta across all subscale categories (Table 3). In study 2, no significant difference in mARM subscale scores were seen from weeks 3 to 7 (refer to Table 3 for subscale scores). Overall, at the individual level, positive correlations (ρ=0.27-0.84) were observed between subscale scores at week 3 and those at week 7 (Table S3 in Multimedia Appendix 1).

**Table 3:** Evaluation of digital working alliance formation at week 3 and digital working alliance maintenance at week 7 as assessed by mobile Agnew Relationship Measure in study 1 and study 2.

mARM^a^ scores			DWA^b^ formation (week 3)				DWA^b^ maintenance (week 7)
			Study 1, mean (SD) (n=40)	Study 2, mean (SD) (n=45)		Study 2, mean (SD) (n=44)	
Overall			5.16 (0.77)	5.36 (1.06)		5.48 (0.97)	
**Subscale scores**							
	Bond	5.67 (1.03)		5.63 (1.31)	5.82 (1.19)		
	Partnership	4.83 (0.72)		5.67 (1.32)	5.85 (1.18)		
	Confidence	5.49 (1.04)		5.68 (1.27)	5.62 (1.23)		
	Openness	4.94 (1.06)		4.83 (1.25)	5.16 (1.04)		
	Client initiative	4.56 (0.75)		4.60 (0.99)	4.64 (0.93)		

^a^mARM: mobile Agnew Relationship Measure.

^b^DWA: digital working alliance.

### Correlations Between DWA and ENS Severity, Engagement, Age, and Race

In both studies, the strength of DWA at week 3, as assessed by the mean overall mARM score, was similar in participants with severe and moderate ENS (Table 4). In study 1, this finding was consistent at each week across the 3-week study. Accordingly, there was no significant correlation between mARM overall scores at week 3 and the baseline CAINS-MAP total score in study 1 (ρ=–0.16, *P*=.36) and study 2 (ρ=–0.13, *P*=.41; Table S4 in Multimedia Appendix 1).

**Table 4:** Evaluation of digital working alliance as assessed by mobile Agnew Relationship Measure overall and subscale scores in people with moderate and severe negative symptoms on week 1, 2, and 3 in study 1 and week 3 in study 2.

mARM^a^ scores		Study 1^b^										Study 2			
		Week 1			Week 2			Week 3			Week 3				
		Moderate (n=29), mean (SD)	Severe (n=10), mean (SD)	Moderate (n=30), mean (SD)		Severe (n=10), mean (SD)	Moderate (n=30), mean (SD)		Severe (n=10), mean (SD)	Moderate (n=40), mean (SD)				Severe (n=4), mean (SD)	
Overall		5.21 (0.75)	4.96 (0.73)	5.19 (0.82)		5.05 (0.82)	5.21 (0.79)		5.00 (0.73)	4.81 (0.94)				4.99 (0.70)	
**Subscale scores**															
	Bond	5.61 (0.95)	5.14 (1.10)	5.72 (1.16)		5.44 (0.82)	5.76 (1.02)		5.40 (1.05)	5.59 (1.37)				6.00 (0.95)	
	Partnership	4.90 (0.82)	4.84 (0.67)	4.80 (0.94)		4.94 (0.61)	4.83 (0.78)		4.84 (0.55)	4.56 (0.93)				4.83 (1.40)	
	Confidence	5.65 (0.95)	5.29 (0.96)	5.55 (0.92)		5.34 (1.06)	5.52 (1.06)		5.40 (1.01)	4.63 (1.01)				4.50 (0.62)	
	Openness	4.97 (1.06)	4.65 (1.23)	5.03 (1.10)		4.85 (1.13)	5.05 (1.13)		4.60 (0.77)	4.21 (1.10)				4.19 (0.90)	
	Client initiative	4.57 (0.88)	4.65 (0.83)	4.57 (0.75)		4.38 (0.78)	4.60 (0.62)		4.43 (1.07)	4.96 (1.16)				5.60 (1.46)	

^a^mARM: mobile Agnew Relationship Measure.

^b^To minimize participant burden, mARM was assessed at weeks 1 and 2 in study 1 only. Severity of negative symptoms based on CAINS-MAP score: moderate <29 points; severe ≥29 points.

**Figure 4:**
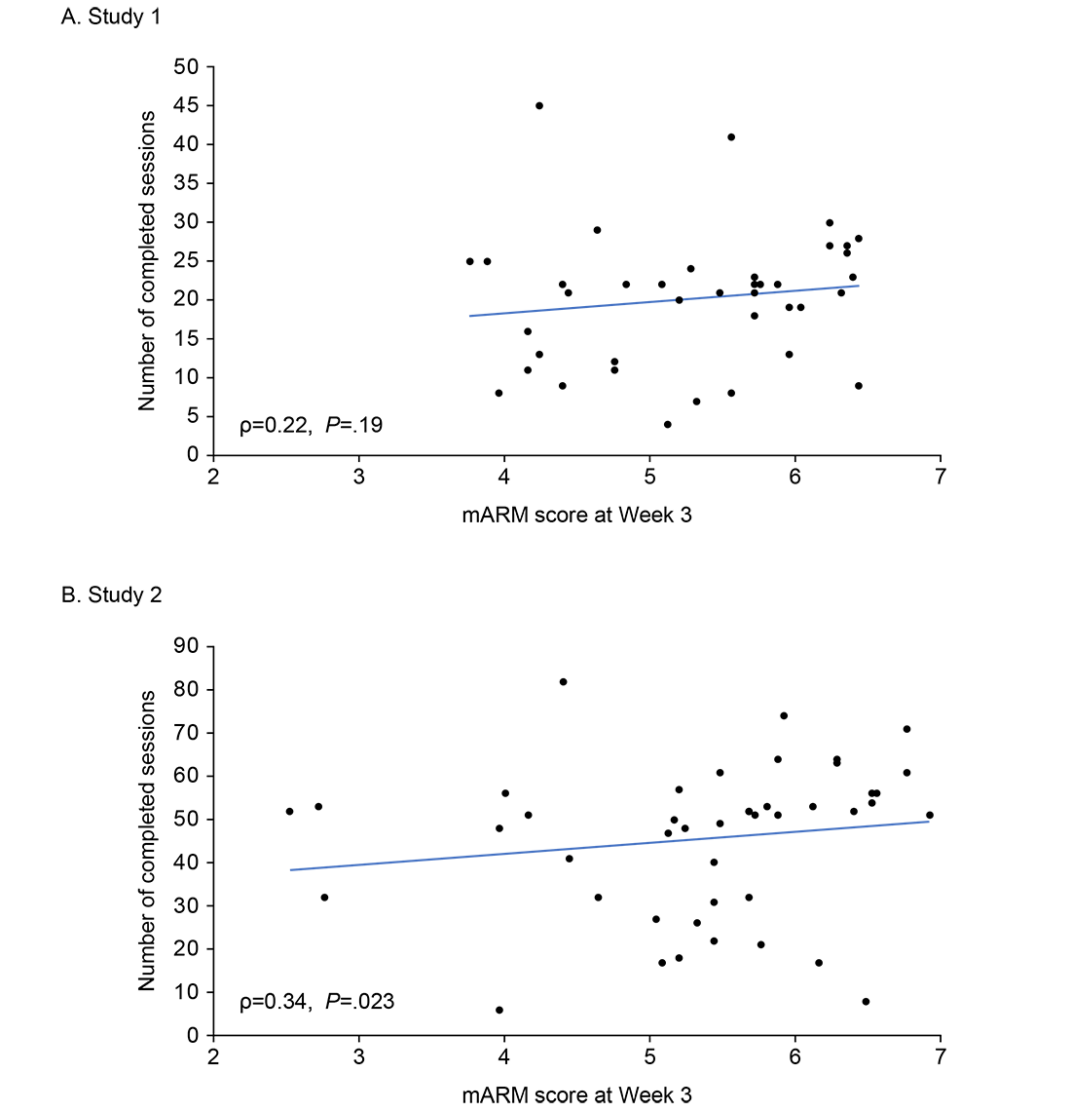
Correlations of mARM overall score at week 3 with number of sessions completed. mARM: mobile Agnew Relationship Measure; ρ: Spearman correlation coefficient.

Strength of DWA correlated positively with the number of completed sessions by participants at week 3 in study 2 (ρ=0.34, *P*=.02), but was not significantly correlated in study 1 (ρ=0.22, *P*=.19; Figure 4). Neither age (study 1, ρ=0.31, *P*=.05; study 2, ρ=0.15, *P*=.32) nor race significantly correlated with overall mARM scores (study 1, *F* test=1.1928, *P*=.72; study 2, *F* test=1.1615, *P*=.75; Table S4 in Multimedia Appendix 1). In this study population, there was no clear relationship between digital literacy and DWA scores. Assessed by quartile MDPQ scores, mean mARM scores in the highest and lowest quartiles were study 1: 4.9 (SD 0.9) and 5.7 (SD 0.4) and study 2: 5.8 (SD 0.9) and 4.8 (SD 1.2), respectively (Table S5 in Multimedia Appendix 1).

### Safety Data Across Two Studies

No ADEs, AEs, or serious AEs were reported during study 1. In study 2, three AEs were reported by 2 participants, none of which were considered severe or related to CT-155 beta use, nor led to study discontinuation. AEs reported were a sinus infection (n=1), arthralgia (n=1), and a rash (n=1). The sinus infection and arthralgia resolved after 6 days; the rash remained present at end of follow-up.

## Discussion

Our findings from 2 independent single-arm multicenter exploratory studies show that a DWA can be established and maintained between people with schizophrenia and a smartphone-based digital therapeutic, namely the abbreviated beta version of CT-155/BI 3972080, a prescription digital therapeutic to target ENS adjunctive to standard of care treatments for schizophrenia. During the orientation phase in study 1, participants established a positive DWA with CT-155 beta. This was replicated in a separate and independent cohort of participants at the end of an equivalent orientation phase in study 2. Furthermore, the positive DWA was maintained during the additional 4-week active phase in study 2, as demonstrated by an equivalent positive mARM score at week 7.

While a strong therapeutic alliance is linked to successful clinical outcomes for in-person therapy, the relationship between DWA, engagement, and outcomes requires further investigation [2-6]. The concept of a therapeutic alliance with DTx is gaining traction in line with the rapid expansion of digital mental health research and implementation; however, the role and relevance of the bond, a core feature of the in-person therapeutic alliance, is somewhat unclear for DTx [40] and not investigated in sufficient detail. Our results add to a growing body of evidence demonstrating that a therapeutic alliance can be established between people with serious mental illnesses and DTx. For example, in recent studies evaluating the benefits of conversational agents (or chatbots) that deliver cognitive behavioral therapy for symptoms of depression, anxiety, and substance use disorder, participants reported high acceptability and affective bond formation [64-67]. Another investigation of a digital health intervention for people with early psychosis, based on cognitive behavioral therapy principles and delivered through a smartphone app, demonstrated that engagement was significantly and positively associated with therapeutic alliance, as assessed by mARM [68]. However, measures to assess bond formation varied with a short-revised version of the Working Alliance Inventory and a Working Alliance Questionnaire (based on the Working Alliance Inventory) being used, making comparisons across studies difficult.

Although there are several lines of evidence demonstrating that a therapeutic alliance is possible for people with schizophrenia and is associated with improved outcomes, there are some factors related to the disorder itself, such as negative symptoms, lack of insight and self-stigma, that can adversely impact the therapeutic alliance between patient and therapist [69-71]. One could therefore speculate that patients who formed and valued a therapeutic alliance with a DTx may accept this additional therapeutic modality for the treatment of schizophrenia. Notably, in our studies, there was no correlation between the severity of negative symptoms at baseline and the establishment or maintenance of a DWA, indicating that even the participants with the most severe symptoms could form and maintain an alliance with CT-155 beta. In line with previous reports examining associations between age and therapeutic alliance we also did not observe any correlation between age and the DWA strength [11,39,71,72]. Similarly, we did not observe any correlation between race and strength of DWA. While some reports emphasize the importance of ethnic matching between patients and clinicians in improving therapeutic alliance [73,74], the lack of correlation with race here highlights the adaptable nature of a DTx modality to fit the needs of different communities.

Although not specifically assessed, the user-centered design process that guided the development of CT-155/BI 3972080 could have improved overall participant engagement and effectiveness. A recent systematic review showed that making end-users central to the product development process leads to enhanced cultural sensitivity, increased acceptance and engagement [75-77]. The review also provided some key recommendations for coproduction, including stakeholder involvement at all stages of development, which was implemented into the development of CT-155/BI 3972080. Considering the 3 pillars of the therapeutic alliance involve enhancing the patient-provider bond, shared goals and adopting a collaborative approach in the therapeutic approach, the patient-centered development process may have contributed to the establishment of a positive DWA achieved here.

The participants in both studies demonstrated a reasonable level of mobile device proficiency. This finding is aligned with recent data from the United States showing that 70%-80% among people with severe mental illness own a smartphone [78-80]. However, smartphone ownership does not necessarily equate with digital literacy [81]. In this study we confirmed that participating people with schizophrenia owning a smartphone showed digital literacy similar to that from the general population [82], but greater than that reported for people with serious mental illness [83]. Indeed, a study in the United Kingdom showed that 42.2% of people with serious mental illness had no foundation digital skills [83], while electronic health literacy has been described as either moderate or low in 2 independent cohorts of adults with schizophrenia spectrum disorders [84]. However, one should consider that the younger generation diagnosed with severe mental illness and schizophrenia are now digital natives and past research may not reflect current trends.

Limitations of these studies include their small sample size, single-arm design, and exploratory nature. While the sample size was limited, the demographics of participants were reflective of the general schizophrenia population including a higher incidence in males [85,86], the majority being Black or African American [87] and a level of education no greater than high school [88]. In addition, almost all (94%) participants in this study reported an annual income under US $25,000, which likely reflects low employment rates in people with schizophrenia [16]. Furthermore, while all participants met inclusion criteria, there was a difference in median MAP-SR score at screening between study 1 and 2. However, as no correlation between ENS severity and DWA was observed in both studies, the difference in baseline MAP-SR scores could be linked to broader population compared in 2 studies. While only a few out of protocol window deviations were recorded, they may impact the DWA assessments over time through asking questions and rapport-building. Preliminary data from these studies support the further evaluation of CT-155 in an ongoing large phase III trial (CONVOKE; NCT05838625). Although the mARM assessment possesses good face and content validity [59], further validation of this assessment for the measurement of DWA in severe chronic mental illness is essential to inform further optimization of this assessment for use in clinical trials [89]. In addition, establishment and assessment of therapeutic alliance between participants and digital devices or apps is relatively new. There is a need to use standardized and validated scales with psychometric validation to assess DWA in future studies which will also make it feasible to compare data and outcomes. Finally, it should also be noted that the eligibility criteria for the studies mandated that participants had a smartphone, an email address, and regular access to the internet through mobile data plan, Wi-Fi or both. Accordingly, the association of digital literacy measured by MDPQ with DWA and engagement should be interpreted in the context of this study population. The study inclusion criteria for participants to own their own mobile device may have biased toward a more digital proficient study population and therefore not reflective of patients who do not own a mobile device and who potentially may have a lower device proficiency. As such, future studies are warranted to understand the impact of lower digital proficiency on adherence to a digital therapeutic and on establishing DWA. While recent evidence suggests that rates of smartphone ownership among adults with severe mental illness (including schizophrenia) are comparable to the general adult US population [78,80], it is encouraging to see high levels of mobile device proficiency in people living with schizophrenia and owning smartphones.

The results from these 2 studies confirm that a positive DWA can be formed within a 3-week period and this DWA was maintained for the entire 7-week duration of study 2, demonstrating that a therapeutic alliance can be formed with a DTx in people living with schizophrenia. Importantly, the formation of a positive DWA was not limited by severe ENS, supporting its potential benefits for those experiencing severe symptoms. Furthermore, people with schizophrenia who participated in these studies demonstrate good digital literacy and acceptance of CT-155 beta for the treatment of ENS. The development of a DTx with proven acceptability, safety, and effectiveness profiles as an adjunctive treatment for ENS of schizophrenia would represent a promising, accessible, scalable, and affordable treatment option for clinical and patient communities for whom the lack of effective treatments for ENS has presented significant challenges. These preliminary data support further evaluation of the potential importance of the patient-CT-155 DWA to therapeutic effectiveness in an ongoing large phase III trial (CONVOKE; NCT05838625). With the expansion of mobile device ownership, DTx offers a potential patient-centered, evidence-based therapeutic support 24 hours a day, 7 days a week. In addition, incorporated into standard of care, evidence based DTx may act as clinician extenders, increasing the breadth and depth of care provided. Understanding how best to engage and optimally implement DTx with people living with schizophrenia to have the greatest reach and impact is of great importance and may shape future clinical research. In addition, understanding who may benefit from a DTx, how to best introduce these tools to patients as part of the clinical workflow, and how to support continued engagement, are important future steps.

## Appendix

Multimedia Appendix 1 Supplementary methods and tables

## Abbreviations

ADE: adverse device effect
AE: adverse event
CAINS-MAP: Clinical Assessment Interview for Negative Symptoms Motivation and Pleasure subscale
DSM-5: Diagnostic and Statistical Manual of Mental Disorders (Fifth Edition)
DTx: digital therapeutics
DWA: digital working alliance
ENS: experiential negative symptoms
FDA: US Food and Drug Administration
ICD-10: International Classification of Diseases, Tenth Revision
MAP-SR: Motivation and Pleasure scale – Self Report
mARM: mobile Agnew Relationship Measure
MDPQ: Mobile Device Proficiency Questionnaire

## Data Availability

To ensure independent interpretation of clinical study results and enable authors to fulfill their role and obligations under the ICMJE (International Committee of Medical Journal Editors) criteria, Boehringer Ingelheim grants all external authors access to clinical study data pertinent to the development of the publication. In adherence with the Boehringer Ingelheim Policy on Transparency and Publication of Clinical Study Data, scientific and medical researchers can request access to clinical study data when it becomes available on Vivli - Center for Global Clinical Research Data [90], and earliest after publication of the primary manuscript in a peer-reviewed journal, regulatory activities are complete, and other criteria are met. Please refer to “Medical & Clinical Trials | Clinical Research | MyStudyWindow” [91] for further information.
