# Supplementary material for: Establishment and Maintenance of a Digital Therapeutic Alliance in People Living With Negative Symptoms of Schizophrenia: Two Exploratory Single-Arm Studies
Source: JMIR Ment Health. 2025 Jan 27;12:e64959. doi: 10.2196/64959 (PMC11811661; doi:10.2196/64959)
Supplement: Multimedia Appendix 1 [file mental_v12i1e64959_app1.docx]

# Supplementary Methods

## Patient-centered design process

An iterative patient-centered design process informed the development of study applications (apps) in Study 1 and 2 and consisted to two phases.

**Exploratory & Generative Phase 1:** This phase involved semi-structured interviews with 4 peer support specialists with lived experiences of schizophrenia, and 15 patient panel participants with a diagnosis of schizophrenia recruited through a patient-owned cooperative platform. The interviews were used to: 1) understand expectations of using a mobile app designed to treat symptoms of schizophrenia, 2) understand potential drivers and barriers to engagement with a digital therapeutic for someone diagnosed with schizophrenia, 3) collect feedback on concepts in development, including but not limited to content, narrative, information architecture, and usability. Concepts were presented using a combination of mockups and clickable prototypes, and interviews were conducted remotely using video-call platforms.

**Generative & Evaluative Phase 2:** This phase involved using the insights from Phase 1 to create a beta app (“study app”) for testing within Study 1. Patients with schizophrenia recruited for Study 1 completed a 1-hour exit interview after using the study app for 3 weeks. The qualitative insights from these interviews helped understand: 1) experience using the study app, 2) drivers and barriers to engagement with the study app, 3) opportunities for usability and program journey improvements, 4) perceived value of specific therapeutic elements within the study app. In tandem with Study 1 exit interviews, a combination of generative and evaluative semi-structured interviews continued to be conducted with the aforementioned 4 peer support specialists and 15 patient panel participants, which were used to collect qualitative feedback on iterative concepts in development.

Patients’ feedback from Study 1 helped informed the development/design of CT-155 app for Study 2. In addition, a similar patient-centered approach was used in Study 2 where all enrolled patients completed a 1-hour exit interview after using the study app for 7 weeks. The insights gathered helped further inform the development of the CT-155 app.

# Supplementary Tables

## Supplementary Table 1: Eligibility criteria for Study 1 (CT-155-C-001) and Study 2 (CT-155-C-002/NCT05486312)

| Eligibility criteria for Studies 1 and 2 |
| --- |
| Inclusion criteria |
| A participant was eligible for entry into the study if all the following inclusion criteria were met:   - Was willing and able to provide written informed consent to participate in the study, attend study visits, and comply with study-related requirements and assessments - Was between 18 and 64 (Study 1) or ≥18 (Study 2) years of age at the time of informed consent - Was fluent in written and spoken English, confirmed by ability to read and understand the informed consent form - Had a primary diagnosis of schizophrenia using the diagnostic criteria for schizophrenia as defined in the International Classification of Diseases, Tenth Revision (ICD-10; Study 1 only) and Diagnostic and Statistical Manual of Mental Disorders, fifth edition (DSM-5; Studies 1 and 2), for at least 1-year prior to screening. - Was in the stable phase of illness, as assessed by the investigator after review of medical records or documented discussion with the treating physician. - Had outpatient treatment status at the time of screening, with no inpatient treatment for schizophrenia within 12 weeks prior to screening. - Was on a stable dose of antipsychotic medication(s) for at least 12 weeks prior to enrolment (Day 1), as determined by the investigator - Had obtained a score of 30 or less on the Motivation and Pleasure – Self Report, as assessed at the screening visit - Was the sole user, per participant self-report, of an iPhone with an iPhone operating system (iOS) 13 or greater or a smartphone with an Android operating system 9 or greater and was willing to download and use CT-155 beta as required per the protocol. - Was the owner of, and had regular access to, an email address - Had regular access to the internet via mobile data plan and/or Wi-Fi - Had stable housing and had remained at the same residence for at least 12 weeks prior to screening, with no anticipated housing changes during the duration of the study - Understood how to use CT-155 beta during the screening visit, as assessed by the investigator during in-clinic installation and activation activities |
| Exclusion criteria |
| A participant was not eligible for study entry if any of the following exclusion criteria were met:   - Was concurrently being treated with more than 2 antipsychotic medications (including more than 2 dosage forms) - Was concurrently being treated with clozapine or haloperidol - Had active prominent positive symptoms that, in the opinion of the investigator, would preclude effective engagement in treatment for negative symptoms - Was concurrently receiving or had received psychotherapy within 12 weeks prior to screening - Met either the International Classification of Diseases or DSM-5 criteria for diagnoses not under investigation, including schizophreniform, schizoaffective, or psychosis non-specific disorders - Had post-traumatic stress disorder, bipolar disorder, major depressive disorder, developmental disorders, or any prominent disorder that would interfere with compliance to the protocol, per investigator judgment - Had substance or alcohol use disorder (excluding caffeine and nicotine), that would interfere with compliance to the protocol, per investigator judgement - Needed or was likely to require prohibited concomitant medications and/or therapy during the study, as determined by the investigator - Was participating in another clinical study (interventional or observational) involving investigational drugs or devices - Had suicidal ideation or behavior, as assessed by the Columbia-Suicide Severity Rating Scale (C-SSRS):   a. Participant that had a “yes” response to either Items 4 or 5 on the C-SSRS Suicidal Ideation Item within the last 12 weeks prior to screening or at baseline visit  b. Participants who had a “yes” response on the C-SSRS Suicidal Behavior Items within the last 26 weeks prior to screening or at baseline visit  c. Participants who, in the opinion of the investigator, presented a serious risk of suicide   - Showed any evidence of a clinically significant concomitant disease or any other clinical condition that would jeopardize the participant’s safety while participating in the clinical study, as judged by the investigator |

## Supplementary Table 2: Serious protocol deviations

|  | Study 1 | | Study 2 | |
| --- | --- | --- | --- | --- |
|  | Number of major deviations  (m=12) | Participants with ≥1 major deviation  (n=10) | Number of major deviations  (m=1) | Participants with ≥1 major deviation  (n=1) |
| **IP dispensed prior to enrollment** | 6 | 6 | – | – |
| Assessment done out of window | 6 | 6 | – | – |
| **IP interruption** | 2 | 2 | – | – |
| Participant uninstalled Study App prior to Week 4 | 1 | 1^a^ | – | – |
| Participant’s phone reset and uninstalled Study App on Day 6 | 1 | 1 | – | – |
| **Out of window visit** | 1 | 1 | 1 | 1 |
| **Inclusion criteria deviation** | 3 | 2 | – | – |
| Schizophrenia diagnosis confirmed <1 year before enrollment | 1 | 1^a^ | – | – |
| Antipsychotic added <12 weeks before screening | 1 | 1^a^ | – | – |
| Individual was ineligible for study but study app was activated, termed an early termination | 1 | 1 | – | – |

^a^One participant in study 1 had 3 serious protocol deviations.

## Supplementary Table 3: Correlations of mARM subscale score at Week 3 and Week 7

| mARM scores, mean (SD) | Week 3 | Week 7 | Correlation between subscale scores between  Week 3 and Week 7 |
| --- | --- | --- | --- |
| Bond | 5.63 (1.31) | 5.82 (1.19) | ρ=0.55; *P*=.0002 |
| Partnership | 5.67 (1.32) | 5.85 (1.18) | ρ=0.84; *P*<.0001 |
| Confidence | 5.68 (1.27) | 5.62 (1.23) | ρ=0.82; *P*<.0001 |
| Openness | 4.83 (1.25) | 5.16 (1.04) | ρ=0.37; *P*=.017 |
| Client initiative | 4.60 (0.99) | 4.64 (0.93) | ρ=0.27; *P*=.081 |

mARM, mobile Agnew Relationship Measure; ρ, Spearman correlation coefficient.

## Supplementary Table 4: Correlations of mARM overall score at Week 3 with CAINS-MAP (baseline) and MAP-SR, age, and race (at screening)

|  | Study 1 | Study 2 |
| --- | --- | --- |
| CAINS-MAP | ρ=−0.16  *P*=.36 | ρ=−0.13  *P*=.41 |
| Age | ρ=0.31  *P*=.05 | ρ=0.15  *P*=.32 |
| Race | F-test=1.1928  *P*=.72 | F-test=1.1615  *P*=.75 |

CAINS-MAP, Clinical Assessment Interview for Negative Symptoms Motivation and Pleasure subscale; mARM, mobile Agnew Relationship Measure; ρ, Spearman correlation coefficient.

## Supplementary Table 5: Assessment of mARM overall score at Week 3 with baseline MDPQ (by quartile)

| MDPQ quartile | Study 1 | | Study 2 | |
| --- | --- | --- | --- | --- |
|  | N with mARM data | Mean (SD) mARM score | N with mARM data | Mean (SD) mARM score |
| 1 (lowest) | 8 | 5.7 (0.4) | 12 | 4.8 (1.2) |
| 2 | 11 | 5.4 (0.8) | 12 | 5.4 (1.1) |
| 3 | 10 | 5.2 (1.0) | 11 | 5.6 (0.8) |
| 4 (highest) | 10 | 4.9 (0.9) | 9 | 5.8 (0.9) |
